# Relapsed breast adenorcarcinoma presenting as pulmonary lymphangitic carcinomatosis

**DOI:** 10.1186/1753-6561-6-S4-P16

**Published:** 2012-07-09

**Authors:** KP Loh, H Ghorab, S Kosuri, M Shah

**Affiliations:** 1 Royal College of Surgeons in Ireland; 2 New York Presbytarian Hospital - Weill Cornell Medical College, USA

## Introduction

Pulmonary lymphagitic carcinomatosis is an entity referring to diffuse infiltration and obstruction of pulmonary parenchymal lymphatic channels by a tumour. The most common malignancies involved are the breasts, lung, colon and stomach. Previous studies have shown that at autopsy up to 24% of patients who died of metastatic breast cancer had pulmonary lymphagitic spread. However the diagnosis of lymphangitic carcimatosis can be challenging due to its non-specific symptoms which include dypsnoea and cough, which occur in a variety of lung diseases.

## Case report

We describe a case of a 84-year old woman who presented with a 9 months history of progressive exertional dyspnoea with occasional productive cough. She was subsequently diagnosed with pulmonary embolism and chronic obstructive pulmonary disease. However despite treatment her dypsnoea had progressed to orthopnoea. In addition, she had a significant past medical history of non-metastatic breast ductal adenocarcinoma diagnosed 19 years ago for which she underwent a right mastectomy as well as being put on tamoxifen for 5 years, she was also diagnosed with tuberculosis 70 years ago. On physical examination, she required 4L of oxygen but was otherwise haemodynamically stable. There were decreased breath sounds at the lung bases bilaterally but more prominent on the right. In addition, the right middle and lower lobes of the lung were dull on percussion. Finally auscultation revealed fine crepitations at the lung bases bilaterally. In terms of investigations her CXR and CT-PE demonstrated a pleural effusion. She then underwent thoracocentesis, pleural biopsy, pleurodesis and right wedge resection. Pathology examination revealed malignant cells stained positive for estrogen receptor (ER), progesterone receptor (PR) and Gross Cystic Disease Fluid Protein-15 (GCDFP15) confirming lymphangitic spread of breast adenorcarcinoma to the lung parenchyma. She was treated with paclitaxel and corticosteroids for palliative purpose.

## Conclusions

In summary, pulmonary lymphangitic carcinomatosis is a common entity in patients with a history of breast carcinoma. Clinical awareness and accurate diagnosis with pathology will guide appropriate treatment and improve the quality of life of the patients. In this patient, her dypnoea is highly likely to be multifactorial although chemotherapy has significantly improved her breathing.

